# Effects of forage NDF strategies on growth performance, starch digestion, ingestive behavior, and inflammatory responses in feedlot Angus × Nellore bulls

**DOI:** 10.1093/tas/txag121

**Published:** 2026-08-07

**Authors:** Mailza Gonçalves de Souza, Jonas Gabriel Dantas, Bianca Lombardi Soares, Matheus Prestes Barros, Matheus Copeli Garcia, Paulo César Bortoleto Júnior, Dante Pazzanese Duarte Lanna

**Affiliations:** University of São Paulo - “Luiz de Queiroz” College of Agriculture, 9, Piracicaba, São Paulo 13418900, Brazil; University of São Paulo - “Luiz de Queiroz” College of Agriculture, 9, Piracicaba, São Paulo 13418900, Brazil; University of São Paulo - “Luiz de Queiroz” College of Agriculture, 9, Piracicaba, São Paulo 13418900, Brazil; University of São Paulo - “Luiz de Queiroz” College of Agriculture, 9, Piracicaba, São Paulo 13418900, Brazil; Department of Animal Science, School of Animal Science and Food Engineering, University of São Paulo, Pirassununga, São Paulo 13635-900, Brazil; Department of Animal Science, School of Animal Science and Food Engineering, University of São Paulo, Pirassununga, São Paulo 13635-900, Brazil; University of São Paulo - “Luiz de Queiroz” College of Agriculture, 9, Piracicaba, São Paulo 13418900, Brazil

**Keywords:** beef cattle, forage neutral detergent fiber, feedlot nutrition, taurine × zebu crossbred

## Abstract

This study evaluated the effects of forage neutral detergent fiber (fNDF) level and feeding strategy on growth performance, carcass traits, ingestive behavior, starch utilization, and inflammatory biomarkers in Angus × Nellore bulls fed energy-dense diets. Sixty Angus × Nellore bulls (initial BW = 398 ± 10 kg) were housed in four pens (15 bulls/pen) and individually monitored using GrowSafe and Intergado systems. During d 0 to 50, bulls received diets containing either 12% or 7% fNDF. During d 51 to 100, 30 bulls remained on their original diets, whereas the remaining 30 bulls were switched to the opposite fNDF level (12→7% or 7→12%). Dry matter intake (DMI), average daily gain (ADG), and feed efficiency did not differ during the first period or across the overall feedlot period (*P* > 0.05). However, during the second period, bulls fed 7% fNDF exhibited greater ADG, improved feed efficiency, greater calculated energy requirements for gain (Eg; Mcal/d), greater performance-predicted dietary net energy for maintenance (NEm; Mcal/kg DM), greater performance-predicted dietary net energy for gain (NEg; Mcal/kg DM), and lower DMI required for maintenance than bulls fed 12% fNDF (*P* = 0.01). In contrast, bulls fed 12% fNDF allocated a greater proportion of DMI to maintenance requirements (*P* = 0.01). Feeding 7% fNDF increased fecal starch concentration and reduced estimated starch digestibility (*P* < 0.05), whereas increasing fNDF from 7% to 12% during the second period improved starch utilization compared with both the continuous 7% fNDF treatment and the 12→7% strategy (*P* = 0.01). Carcass traits, water intake, and the frequency of visits to the water trough were not affected by treatment (*P* > 0.05). Diets containing 12% fNDF increased chewing and rumination time during the first period (*P* < 0.05), whereas reducing fNDF, particularly following the 12→7% transition, decreased rumination time and increased idle time without affecting eating time (*P* < 0.05). Concentrations of lactate dehydrogenase, lipopolysaccharide (LPS), and haptoglobin were not affected by treatment *(P* > 0.05), whereas serum amyloid A (SAA) concentrations increased in bulls fed 7% fNDF during the second period (*P* = 0.01). The 7% fNDF strategy improved late-feedlot growth performance but at the expense of starch utilization, as evidenced by greater fecal starch concentration and lower estimated starch digestibility. In contrast, increasing fNDF during the finishing phase improved starch utilization without compromising animal performance. Although these responses were evident during the second period, they did not result in detectable differences in overall feedlot growth performance.

## Teaser

Sequential modulation of forage fiber alters feeding behavior and starch utilization without compromising performance in feedlot crossbred bulls.

## Introduction

Crossbreeding *Bos taurus* (Aberdeen Angus) with *Bos indicus* (Nellore) has become an important strategy approach in feedlot production systems to enhance productivity and reduce days on feed. Angus × Nellore cattle combine the superior growth potential of taurine breeds with the heat tolerance and adaptability of zebu cattle, providing complementary advantages under tropical intensive production systems (Amaral et al. 2018). Previous studies have reported physiological and digestive differences between purebred Nellore and taurine × zebu crossbred cattle, including variation in ruminal retention time, passage rate, and fermentative capacity, highlighting the need for genotype-specific nutritional strategies (Phillips et al. 1960; Oliveira et al. 1991; Hegarty 2004).

Dietary fiber plays a central role in maintaining rumen function by promoting chewing activity, stabilizing ruminal fermentation, and supporting microbial function (Van Soest et al. 1991; Van Soest 1994; Allen 1997). In energy-dense feedlot diets, relatively small changes in forage neutral detergent fiber (fNDF) concentration can influence ingestive behavior, ruminal function, and animal performance (Pitt et al. 1996; Castillo-Lopez et al. 2014). Therefore, adequate fNDF is essential to maintain ruminal health and productive efficiency (NASEM 2016; Shi et al. 2023).

Conversely, greater reliance on concentrate feeds and reduced forage neutral detergent fiber (fNDF) concentrations have been associated with increased ruminal endotoxin production and elevated circulating inflammatory biomarkers, including haptoglobin, serum amyloid A, lipopolysaccharide (LPS), and lactate dehydrogenase (LDH), suggesting an increased risk of systemic inflammation in feedlot cattle (Zebeli et al. 2012). Previous studies have shown that increasing forage inclusion or dietary fNDF concentration can improve energy utilization and weight gain in feedlot cattle (Defoor et al. 2002; Galyean and Defoor 2003). In Brazil, studies evaluating different forage sources, including raw sugarcane bagasse (Leme et al. 2003; de Melo et al. 2019) and sugarcane silage (Caetano et al. 2015), have identified optimal fNDF levels ranging from approximately 11.3% to 13.7% of dietary dry matter, depending on grain processing method and forage characteristics. However, these recommendations were developed primarily for zebu cattle, limiting their direct application to Angus × Nellore bulls.

In contrast to research-based recommendations, commercial Brazilian feedlots often use lower forage inclusion rates. Monsalve and Millen (2025) reported an average forage inclusion of 15.7% of dietary dry matter, reflecting the widespread adoption of high-concentrate finishing diets that frequently result in fNDF concentrations below those evaluated in experimental studies (Zinn et al. 2004; Caetano et al. 2015). Furthermore, fiber requirements are unlikely to remain constant throughout the feedlot period, as they may change according to animal growth stage, intake patterns, and ruminal adaptation over time (Pitt et al. 1996). Despite the increasing adoption of Angus ×Nellore bulls in Brazilian feedlots, information regarding optimal fNDF strategies for this genotype remains limited. Consequently, it remains uncertain whether current recommendations, which are largely based on studies with other cattle populations, are appropriate for Angus × Nellore bulls given their distinct digestive physiology and ingestive behavior relative to purebred Nellore cattle (Hegarty 2004).

Based on the average forage inclusion used commercially in Brazil (15.7%; Monsalve and Millen 2025) and the typical NDF concentration of corn silage (∼50%; Oliveira et al. 2017), a practical high-concentrate finishing diet would provide approximately 7% fNDF, whereas 12% fNDF corresponds to the experimentally determined optimum reported by Caetano et al. (2015). We hypothesized that strategically adjusting in dietary fNDF throughout the feedlot period could optimize the balance between growth performance, starch utilization, and ruminal function in Angus × Nellore bulls. To test this hypothesis, we compared constant dietary fNDF concentrations (12% or 7%) with two transition strategies (12→7% and 7→12%) and evaluated their effects on growth performance, digestive and metabolic responses (including fecal starch concentration and estimated starch digestibility), feeding behavior, inflammatory biomarkers, and carcass characteristics in Angus × Nellore bulls finished on energy-dense diets.

## Material and methods

All experimental procedures were conducted in accordance with the guidelines of the Brazilian National Council for the Control of Animal Experimentation and were approved by the Ethics Committee on Animal Use of the Luiz de Queiroz College of Agriculture, University of São Paulo (protocol no. 2833020223; ID 000421). The experiment was carried out at the Nutrigenomics Center of ESALQ/USP, Piracicaba, São Paulo, Brazil.

### Animals and experimental design

The study was conducted from April to July using 60 Angus ×Nellore bulls with an average initial BW of 398 ± 10 kg and an average age of 18 months. All bulls received standard veterinary care (deworming and respiratory and clostridial vaccinations) and were identified with two electronic ear tags (Allflex Livestock Intelligence, Joinville, SC, Brazil) compatible with the Intergado (Betim, MG, Brazil) and GrowSafe (GrowSafe Systems Ltd., Airdrie, AB, Canada) monitoring systems. Animals were housed in four collective pens (10 × 16.75 m; 167.5 m²; 15 bulls/pen), each equipped with two GrowSafe feeding bunks, one Intergado automated weighing system, and one Intergado water trough, allowing individual monitoring of feed intake, body weight, and water visitation. Although each feeding strategy was represented by a single physical pen during the second experimental period, the individual animal remained the experimental unit because all intake, body weight, and behavioral measurements were recorded individually using automated monitoring systems. In addition, experimental groups were rotated among physical pens every 14 d to minimize potential environmental effects associated with pen location.

**Figure 1:**
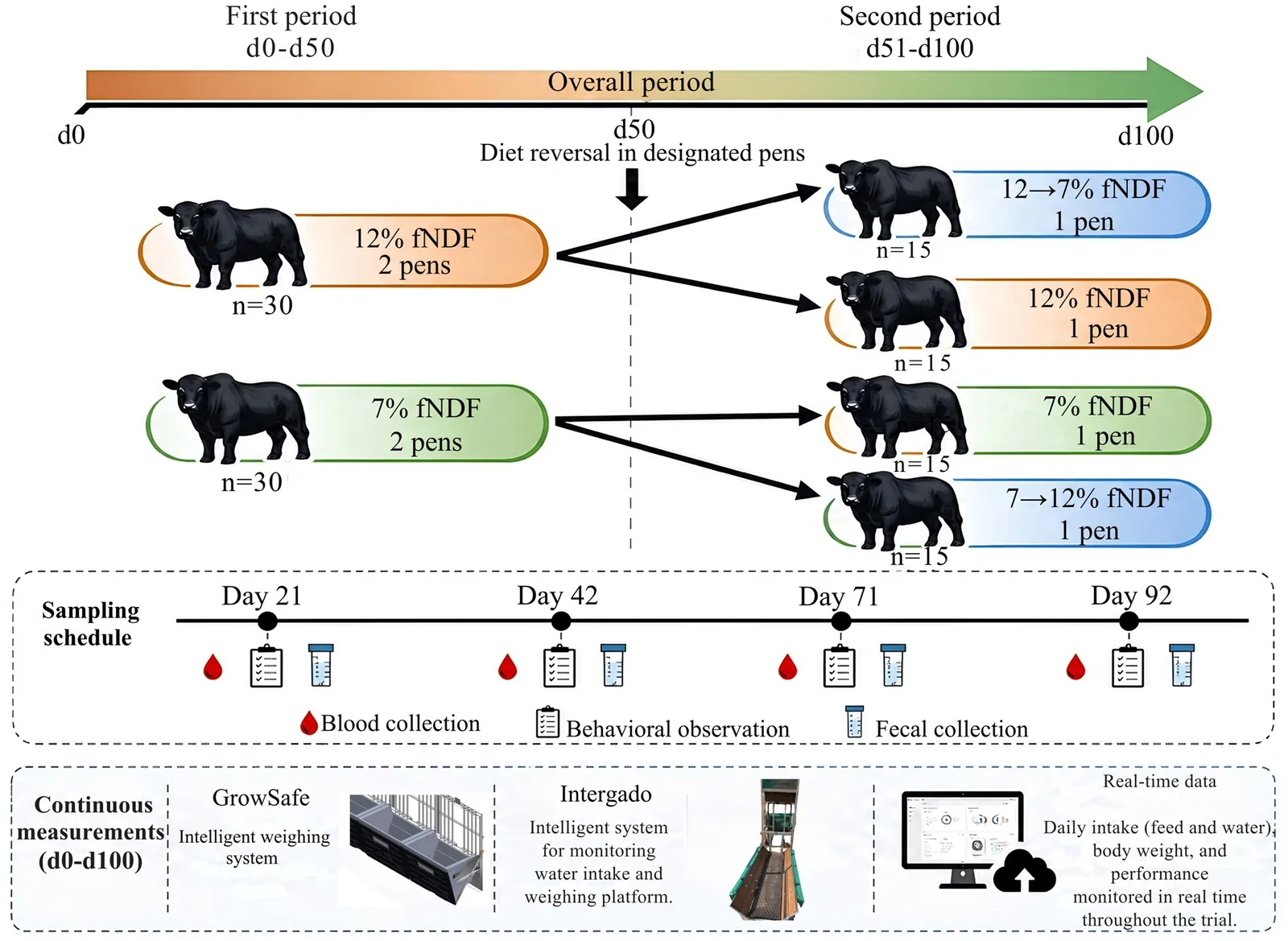
Experimental design and timeline of the trial. The experiment was analyzed for: the first period (d 0 to 50), second period (d 51 to 100), and the 100 days. In the first period, diets had either 12% or 7% neutral detergent fiber from forage on a DM basis (12% or 7% fNDF); 30 bulls in four pens, two per treatment. On day 50, two pens were assigned to a different diet, and two continued with the same diet, resulting in four treatments in the second period (15 bulls per treatment; one pen each): 12% fNDF, 7% fNDF, 12→7% fNDF, and 7→12% fNDF. The design allowed the evaluation of fixed versus changing fNDF levels.

The trial lasted 100 d following a 21-d dietary adaptation period and was divided into Period 1 (d 0 to 50), Period 2 (d 51 to 100), and the overall experimental period (d 0 to 100). During Period 1, bulls received diets containing either 12% or 7% forage neutral detergent fiber (fNDF; DM basis), with two pens assigned to each treatment. During Period 2, one pen from each treatment remained on the same diet, whereas the other pen was switched to the opposite fNDF level (12→7% or 7→12%), resulting in four feeding strategies: constant 12% fNDF, constant 7% fNDF, 12→7% fNDF (step-down), and 7→12% fNDF (step-up) (Fig. 1).

### Analysis of diet components

Ingredient composition of the adaptation and experimental diets is presented in Tables 1 and 2. Experimental diets were formulated using the RLM (Ração de Lucro Máximo) ration formulation software (Piracicaba, SP, Brazil) to meet the nutrient requirements of finishing cattle (Lanna et al. 1999; Ração de Lucro Máximo [RLM] 2021). Samples of feed ingredients from the single ingredient batches used throughout the experiment were collected for determination of dry matter (DM), crude protein, ash, extract, starch, neutral detergent fiber (NDF), and acid detergent fiber (ADF). Dry matter concentration of corn silage was determined daily using a forced-air oven at 105°C (AOAC Method 934.01) to adjust dietary inclusion rates, whereas samples for the remaining chemical analyses were obtained from the single batches of each feed ingredient used during the study. Chemical analyses followed AOAC (1997) procedures. Neutral detergent fiber and ADF were determined according to Van Soest et al. (1991).

**Table 1:** Nutritional composition of ingredients of % DM and diets of adaptation.

Ingredients (%DM^a^)				
Itens	Corn silage	Ground corn	Cottonseed	Soybean meal
**Dry matter**	37.30	88.30	93.10	89.30
**Crude protein**	8.90	8.30	25.40	48.70
**Mineral**	4.20	1.30	3.90	7.00
**NDF^b^**	51.50	10.20	50.40	11.60
**ADF^c^**	26.80	3.40	33.70	6.20
**Starch**	30.80	72.60	–	–
**Ether extract**	3.80	5.00	15.70	2.30
**Diets during the adaptation period**				
**Days**	0–2	3–9	10–16	17–21
**Ingredients (%DM)**				
**Corn silage**	96.80	48.00	28.00	23.99
**Ground corn**	–	35.40	55.40	64.08
**Cottonseed**	–	10.00	10.00	7.00
**Soybean meal**	2.30	3.00	3.00	1.50
**Urea**	0.40	1.30	1.30	1.20
**Premix**	0.50	2.00	2.00	2.08
**Potassium chloride**	–	0.25	0.25	0.25

a DM, Dry matter.

b NDF, Neutral detergent fiber.

c ADF, Acid detergent fiber.

**Table 2:** Composition of experimental diets.

Composition (% DM)^a^	Diets	
	12% fNDF^b^	7% fNDF^b^
**Corn Silage**	23.89	13.90
**Ground Corn**	64.08	73.82
**Cottonseed**	7.00	7.00
**Soybean Meal**	1.50	1.50
**Urea**	1.20	1.20
**Premix^c^**	2.08	2.08
**Potassium Chloride**	0.25	0.50
**Nutritional composition (% of DM)^a^**		
**Dry matter**	66.87	74.70
**Ether extract**	5.24	5.35
**Crude protein**	12.59	12.98
**Rumen degradable protein**	8.48	8.67
**Neutral detergent fiber**	22.25	18.19
**Acid detergent fiber**	11.06	8.68
**Total Digestible Nutrients**	73.78	75.60
**NEm (Mcal/kgMS)**	2.67	2.74
**NEg (Mcal/kgMS)**	1.14	1.19

a DM, Dry matter.

b fNDF, Neutral Detergent Fiber from Forage.

c Premix = Supplemental vitamins and minerals (per kg of mineral supplement): calcium, 230–280 g; phosphorus, 20 g; fluorine (maximum), 200 mg; sodium, 80 g; magnesium, 20 g; sulfur, 25 g; selenium, 10 mg; zinc, 3000 mg; manganese, 1000 mg; copper, 750 mg; cobalt, 20 mg; iodine, 40 mg; vitamin A, 125,000 IU; vitamin E, 500 IU; monensin sodium, 1250 mg.

### Measurement of live weight, water intake, and dry matter intake

Body weight and water intake were obtained using four Intergado system automatic weighing platforms integrated with water troughs. Each pen was fitted with one unit, serving 15 bulls. Each pen was equipped with two GrowSafe feed bunks which recorded daily feed disappearance associated with each steer’s unique radio frequency identification tag to calculate individual animal feed intake. Average daily gain was estimated separately for each animal within each experimental period using the linear regression of body weight against time (days), as described by Ferreiro and Preston (1976). Daily body weight measurements obtained from the Intergado automated weighing system provided dozens of individual observations per animal, allowing the slope of the regression to be used as an estimate of ADG rather than calculating ADG from only the initial and final body weight measurements.

### Diet management

Diets were mixed for 5 min using a mixer wagon (American Calan, Northwood, NH, USA) and offered twice daily: 50% at 0700 h and 50% at 1600 h. Feed was prepared immediately before delivery. Orts were weighed daily to adjust feed allowance and maintain ad libitum intake. Particle size distribution of corn silage and experimental diets was determined using the Penn State Particle Separator according to Lammers et al. (1996) (Table 3). Physically effective neutral detergent fiber (peNDF) was calculated according to Mertens (1997) as dietary NDF concentration multiplied by the proportion of particles retained on sieves ≥ 4 mm.

**Table 3:** Particle size of corn silage and experimental diets.

		Experimental diets	
Item	Corn Silage	12% fNDF^a^	7% fNDF^a^
**19 mm**	5.06	1.00	0.58
**8 mm**	62.56	27.60	16.58
**4 mm**	12.68	5.10	4.24
**Particles less than 4 mm**	19.69	66.40	78.76
**peNDF^b^**	41.68	7.48	3.90

a fNDF, Neutral detergent fiber from forage.

b peNDF, The physically effective NDF (peNDF) of the total diet was calculated as the NDF concentration in the diet multiplied by the proportion of particles retained on sieves ≥ 4 mm using the Penn State Particle Separator (Mertens 1997).

### Performance-predicted dietary net energy

Performance-predicted dietary net energy for maintenance (NEm) and gain (NEg) were estimated according to the equations proposed by Zinn and Shen (1998). Net energy requirements for gain (Eg; Mcal/d) and maintenance (Em; Mcal/d) were first calculated from average daily gain (ADG; kg/d) and shrunk body weight (SBW; kg), assuming SBW = 0.96 × BW, using the following equations:


$$Eg=0.0493\times SBW^{0.75}\times ADG^{1.097}$$

(NRC 1984)


$$Em=0.077\times SBW^{0.75}$$

(Lofgreen and Garrett 1968)

The performance-predicted dietary NEm concentration (Mcal/kg DM) was estimated by solving the quadratic equation described by Zinn and Shen (1998):


$$\mathrm{NEm}=\frac{-b\pm \sqrt{b^{2}-4ac}}{2c}$$

where


a=-0.41×Em

b=0.877×Em+0.41×DMI+Eg

c=-0.877×DMI

where DMI is dry matter intake (kg/d).

Performance-predicted dietary NEg (Mcal/kg DM) was subsequently calculated as:


NEg=0.877×NEm-0.41

(Zinn and Shen 1998).

The values reported in Table 6 represent performance-predicted dietary NEm and NEg concentrations (Mcal/kg DM) estimated from observed animal performance and DMI rather than formulated dietary energy values. The DMI remaining for growth (DMIg) was then calculated as the difference between the total daily intake and the portion allocated to maintenance, as described by Silva (2001).

### Estimation of fecal starch, fecal pH, and starch digestibility

Fecal samples were collected from the rectum on days 21, 42, 71, and 92 for analysis of fecal starch and estimation of apparent starch digestibility; in addition, additional samples were collected for pH determination. Samples were homogenized, dried at 65°C for 48 h, and ground in a ‘Valley’ type mill through a 1-mm sieve. Fecal starch content was estimated by NIR spectroscopy (Foss NIRS ystems Inc., Silver Spring, MD, USA) using the equation of Caetano et al. (2009); pH was measured with a pH meter after collection, and apparent starch digestibility was estimated by the equation of Zinn et al. (2007).

### Ingestive behavior

The bulls were continuously monitored for 24 h during four evaluation periods (at 21, 42, 71, and 92 days), starting after the second feeding at 5:00 pm and ending at the time of the last feeding the following day. During this period, idleness, rumination, and feed intake (minutes/day) were recorded. Observations were made at fixed 10-min intervals, following the methodology described by Goulart et al. (2020a). Total chewing time was determined by the sum of feed intake and rumination time (Maekawa et al. 2002; Kononoff et al. 2003). Based on the recorded values, the following parameters were calculated for each animal: daily feed intake time (min/day), daily rumination time (min/day), daily idle time (min/day), and daily chewing time (min/day) (Maekawa et al. 2002).

### Blood parameters

Blood samples were collected on days 21, 42, 71, and 92. Samples were obtained via jugular venipuncture using 10 mL Vacutainer tubes, centrifuged at 3000 rpm for 10 min at 4°C, and the serum was stored in Eppendorf tubes at −20°C, as described by Kerr (2003), for later analysis. Lactate dehydrogenase was measured using an automated biochemistry system (Model SBA–200, CELM, Barueri–SP, Brazil) with LABTEST Diagnóstica SA kits (Lagoa Santa–MG, Brazil). Haptoglobin concentrations were determined using an ELISA kit following the methods of Makimura and Suzuki (1982) and Cooke and Arthington (2013). Serum amyloid A and LPS were analyzed using a commercial ELISA kit (Bovine Cattle SAA, catalog ELK5620, ELK Biotechnology).

### Carcass evaluations

On the first and last days of the experimental period, carcass characteristics were evaluated by ultrasonography using an ExaGo ultrasound scanner (IMV imaging, Bellshill, Scotland, UK) equipped with a 3.5-MHz linear transducer (180 mm) and an acoustic guide. Images were obtained to evaluate loin eye area (LEA), subcutaneous fat thickness (SFT) between the 12th and 13th ribs, and rump fat thickness (FTR) at the P8 site over the *Biceps femoris muscle*. A good quality image was stored for later analysis and interpretation using the Lince software.

### Statistical analysis

All statistical analyses were performed using SAS University Edition (SAS Institute Inc., Cary, NC). Residuals were evaluated for normality using the Shapiro–Wilk test, and potential outliers were identified with the UNIVARIATE procedure. The experiment was conducted as a completely randomized design. The individual animal was considered the experimental unit (*n* = 15 per treatment) because feed intake, body weight, water intake, and behavioral variables were recorded individually throughout the experiment using the GrowSafe and Intergado automated monitoring systems. Consequently, each animal generated an independent set of response variables.

Although bulls were group-housed, experimental groups were rotated among physical pens every 14 d while remaining on their assigned dietary treatments. This management strategy minimized potential confounding associated with pen location (e.g., shade, solar exposure, and airflow), preventing any physical pen from remaining permanently associated with a given dietary treatment throughout the experiment.

Therefore, pen was treated as a random effect in the statistical model to account for residual environmental variation, whereas treatment effects were evaluated using the individual animal as the experimental unit.

Statistical analyses were conducted using the MIXED procedure. Dietary treatment was specified as a fixed effect in the model. To account for the rotation scheme, physical pen was included as a random effect, representing environmental variation distributed among treatments over time. Bulls nested within treatment were included as a random effect to capture animal-to-animal variability. For analyses involving time intervals (0 to 50, 51 to 100, and 0 to 100 days), each period was evaluated independently using the same modeling framework. Treatment means are reported as least squares means (LSMeans), and statistical significance was declared at *P* ≤ 0.05.

## Results

### Growth performance, dry matter intake, and feed efficiency

Growth performance, DMI, and feed efficiency for the first (d 0 to 50), second (d 51 to 100), and overall (d 0 to 100) periods are presented in Table 4. No differences among treatments were detected during the first period (*P* > 0.05). During the second period, bulls fed the constant 7% fNDF diet had greater ADG than those maintained on the constant 12% fNDF diet, with an improvement of 0.32 kg/d (approximately 20%; *P* = 0.01). Bulls transitioned from 7% to 12% fNDF observed a similar response, whereas those transitioned from 12% to 7% fNDF exhibited intermediate values. Feed efficiency followed a similar pattern, being greater in bulls fed the constant 7% fNDF diet and in those transitioned from 12% to 7% fNDF than in bulls maintained on the constant 12% fNDF diet, representing improvements of approximately 19 and 16%, respectively (*P* = 0.01). Bulls transitioned from 7% to 12% fNDF exhibited intermediate values and did not differ from the other treatments (*P* > 0.05). Dry matter intake did not differ among treatments during any experimental period (*P* > 0.05). Likewise, water intake, frequency of visits to the water trough, and carcass characteristics were not affected by treatment (Tables 4 and 5; *P* > 0.05).

**Table 4:** Growth performance, dry matter intake, and feed efficiency of Angus × Nellore bulls fed different fNDF strategies in the feedlot.

	Treatment					
Item	**12% fNDF^a^^,^** ^b^	**7% fNDF** ^b^ ^,^ ^c^	12→7% fNDF^b^^,^^d^	7→12% fNDF^b^^,^^e^	SEM^f^	*P-*value
**Live weight (kg)**						
**d 0**	396.53	401.40	–	–	2.97	0.41
**d 50**	501.81	503.51	–	–	5.07	0.86
**d 51**	499.90	506.90	494.10	495.70	4.69	0.78
**d 0 to 100**	593.20	604.63	582.46	598.16	6.21	0.67
**DMI (kg/d)^g^**						
**d 0 to 50**	11.36	11.85	–	–	0.18	0.24
**d 51 to 100**	11.51	11.47	11.11	11.78	0.19	0.71
**d 0 to 100**	11.43	11.76	11.54	11.68	0.17	0.91
**ADG (kg/d)^h^**						
**d 0 to 50**	2.14	2.30	–	–	0.05	0.20
**d 51 to 100**	1.61 b	1.93a	1.79ab	1.85a	0.03	0.01
**d 0 to 100**	2.00	2.12	1.90	2.07	0.03	0.23
**Feed efficiency (ADG/DMI)**						
**d 0 to 50**	0.183	0.194	–	–	0.003	0.14
**d 51 to 100**	0.140 b	0.166a	0.162a	0.152ab	0.002	0.01
**d 0 to 100**	0.174	0.180	0.167	0.178	0.002	0.15
**Water intake (kg/day)**	30.20	32.70	27.80	30.60	0.74	0.14
***N*° visits to the water trough**	4.20	4.60	3.80	3.90	0.11	0.08

a 12% fNDF, constant 12% fNDF throughout the 100 d.

b fNDF, Neutral Detergent Fiber from Forage.

c 7% fNDF, constant7% fNDF throughout the 100 d.

d 12→7% fNDF, step-down fNDF, from 12% in period 1% to 7% in period 2.

e 7→12% fNDF, step-up fNDF, from 7% in period 1% to 12% in period 2.

f SEM, Standard error of the mean.

g DMI, Dry matter intake.

h ADG, Average daily gain.

*Means with different superscript letters within a row differ*.

**Table 5:** Carcass characteristics of Angus** × **Nellore bulls fed different fNDF strategies in the feedlot.

	**Treatment**					
Item	12% fNDF^a^^,^^b^	7% fNDF^b^^,^^c^	12→7% fNDF^b^^,^^d^	7→12% fNDF^b^^,^^e^	SEM^f^	*P-*value
**Initial**						
**REA (cm**2**)^g^**	57.92	55.76	57.09	54.16	0.73	0.27
**SF (mm)** ^h^	0.06	0.207	0.109	0.123	0.06	0.89
**FTR (mm)^i^**	0.14	0.70	0.42	0.83	0.13	0.22
**Final**						
**REA (cm**2**)**	85.71	82.9	85.20	85.62	0.87	0.65
**SF (mm)**	6.28	6.89	7.08	6.88	0.19	0.49
**FTR (mm)**	10.24	11.23	10.22	11.40	0.41	0.63

a 12% fNDF, constant 12% fNDF throughout the 100 d.

b fNDF, Neutral Detergent Fiber from Forage.

c 7% fNDF, constant7% fNDF throughout the 100 d.

d 12→7% fNDF, step-down fNDF, from 12% in period 1% to 7% in period 2.

e 7→12% fNDF, step-up fNDF, from 7% in period 1% to 12% in period 2.

f SEM, Standard error of the mean.

g REA, Ribeye area.

h SF, Subcutaneous Fat Thickness.

i FTR, Rump Fat Thickness.

### Energy requirements and performance-predicted dietary net energy

Estimates of energy requirements and performance-predicted dietary net energy for the first (d 0 to 50), second (d 51 to 100), and overall (d 0 to 100) periods are presented in Table 6. No treatment effects were observed during the first or overall periods (*P* > 0.05). During the second period, bulls fed the constant 7% fNDF diet had greater energy required for gain (Eg) than bulls maintained on the constant 12% fNDF diet, with an increase of 2.47 Mcal/d (approximately 25%; *P* = 0.01). Likewise, performance-predicted dietary NEm and NEg were greater in bulls fed the constant 7% fNDF diet, with increases of approximately 13% and 17%, respectively (*P* = 0.01). Conversely, bulls maintained on the constant 12% fNDF diet required approximately 11% more DMI to meet maintenance requirements than bulls fed the constant 7% fNDF diet (*P* = 0.01). No treatment effects were observed for energy required for maintenance (Em) or DMI required for gain (*P* > 0.05).

**Table 6:** Performance-predicted dietary net energy and energy utilization of Angus × Nellore bulls fed different fNDF strategies during the feedlot period.

Item	Treatment					
	12% fNDF^a^^,^^b^	7% fNDF**^b^^,^^c^**	12→7% fNDF^b^^,^^d^	7→12% fNDF^b^^,^^e^	SEM^f^	*P-*value
**Energy required for gain (Eg, Mcal/d)^g^**						
**0 to 50**	11.71	12.86	–	–	2.79	0.14
**51 to 100**	10.02 b	12.49a	11.21ab	11.71ab	2.14	0.01
**0 to 100**	12.76	14.59	12.95	13.65	3.07	0.42
**Energy required for maintenance (Em, Mcal/d)^h^**						
**0 to 50**	8.10	8.13	–	–	0.44	0.81
**51 to 100**	9.22	9.42	9.20	9.30	0.52	0.72
**0 to 100**	9.22	9.42	9.29	9.29	0.5	0.78
**Performance-predicted dietary NEm (Mcal/kg DM)^i^**						
**0 to 50**	2.16	2.26	–	–	0.22	0.11
**51 to 100**	2.08 b	2.36a	2.30a	2.23ab	0.17	0.01
**0 to 100**	2.38	2.51	2.35	2.41	0.22	0.35
**Performance-predicted dietary NEg (Mcal/kg DM)^j^**						
**0 to 50**	1.48	1.57	–	–	0.19	0.10
**51 to 100**	1.42 b	1.66a	1.60a	1.55ab	0.15	0.01
**0 to 100**	1.68	1.79	1.65	1.70	0.19	0.34
**DMI required for maintenance (kg/d)^k^**						
**0 to 50**	3.76	3.61	–	–	0.32	0.11
**51 to 100**	4.44a	3.99b	4.02b	4.19ab	0.39	0.01
**0 to 100**	3.89	3.79	4.00	3.88	0.34	0.51
**DMI required for gain (kg/d)^l^**						
**0 to 50**	7.82	8.12	–	–	1.35	0.40
**51 to 100**	7.08	7.48	6.96	7.61	1.19	0.47
**0 to 100**	7.55	8.17	7.77	7.97	1.25	0.60

a 12% fNDF, constant 12% fNDF throughout the 100 d.

b fNDF, Neutral Detergent Fiber from Forage.

c 7% fNDF, constant 7% fNDF throughout the 100 d.

d 12→7% fNDF, step-down fNDF, from 12% in period 1% to 7% in period 2.

e 7→12% fNDF, step-up fNDF, from 7% in period 1% to 12% in period 2.

f SEM, Standard error of the mean.

g Energy required for gain (Eg), Mcal/d, calculated according to NRC (1984).

h Energy required for maintenance (Em), Mcal/d, calculated according to Lofgreen and Garrett (1968).

i Performance-predicted dietary NEm (Mcal/kg DM), estimated according to Zinn and Shen (1998).

j Performance-predicted dietary NEg (Mcal/kg DM), estimated according to Zinn and Shen (1998).

k Dry matter intake required for maintenance (DMIm), kg/d.

l Dry matter intake required for gain (DMIg), kg/d.

*Means with different superscript letters within a row differ*.

### Fecal pH, fecal starch, and estimated starch digestibility

Fecal pH, fecal starch concentration, and estimated starch digestibility are presented in Table 7. No treatment effects were observed for fecal pH throughout the experimental period (*P* > 0.05). During the first period (d 21), bulls fed the constant 7% fNDF diet exhibited greater fecal starch concentration than those fed the constant 12% fNDF diet, representing an increase of 2.64 percentage units (approximately 35%; *P* = 0.02). Accordingly, estimated starch digestibility was reduced by 1.47 percentage units (approximately 1.5%; *P* = *0.02*). During the second period (d 71), bulls transitioned from 7% to 12% fNDF exhibited the lowest fecal starch concentration, whereas bulls fed the constant 7% fNDF diet and those transitioned from 12% to 7% fNDF exhibited the greatest values (*P* > 0.01). This response was accompanied by greater estimated starch digestibility in bulls transitioning from 7% to 12% fNDF than in bulls maintained on the constant 7% fNDF diet (*P* = 0.02). By d 92, bulls fed the constant 7% fNDF diet had the greatest fecal starch concentration and the lowest estimated starch digestibility among treatments (*P* > 0.01).

**Table 7:** Fecal pH, fecal starch concentration, and estimated starch digestibility of Angus** × **Nellore bulls fed different fNDF strategies in the feedlot.

Item	Treatment					
	**12% fNDF^a^^,^** ^b^	7% fNDF^b^^,^^c^	12→7% fNDF^b^^,^^d^	7→12% fNDF^b^^,^^e^	SEM^f^	*P-*value
**pH fecal**						
**21 d**	6.62	6.37			0.07	0.10
**42 d**	6.60	6.79			0.07	0.21
**71 d**	6.92	7.01	6.87	7.07	0.06	0.73
**92 d**	7.0	6.70	6.96	6.60	0.07	0.32
**Fecal starch (%)**						
**21 d**	7.65b	10.29a			0.57	0.02
**42 d**	5.30	6.62			0.35	0.06
**71 d**	5.17ab	6.82a	6.48a	4.25b	0.28	<0.01
**92 d**	5.61b	10.46a	6.00b	6.35b	0.45	<0.01
**Estimated starch digestibility (%)**						
**21 d**	95.88a	94.41b			0.31	0.02
**42 d**	97.17a	96.44b			0.19	0.05
**71 d**	97.23ab	96.32b	96.53b	97.77a	0.16	0.02
**92 d**	97.00a	94.32b	96.79a	96.59a	0.25	<0.01

a 12% fNDF, constant 12% fNDF throughout the 100 d.

b fNDF, Neutral Detergent Fiber from Forage.

c 7% fNDF, constant 7% fNDF throughout the 100 d.

d 12→7% fNDF, step-down fNDF, from 12% in period 1% to 7% in period 2.

e 7→12% fNDF, step-up fNDF, from 7% in period 1% to 12% in period 2.

f SEM, Standard error of the mean.

### Ingestive behavior

Dry matter intake time, chewing time, rumination time, and idle time are presented in Table 8. Dry matter intake time was not influenced by dietary treatments throughout the experimental period (*P* > 0.05). In contrast, daily chewing time and rumination time were significantly affected by fNDF levels and their transitions (*P* < 0.05). During the first period, bulls fed the 12% fNDF diet exhibited greater chewing and rumination times than those fed the 7% fNDF diet, representing increases of approximately 21% and 33%, respectively (*P* < 0.01). At d 71, bulls subjected to the 12→7% transition exhibited greater chewing time than bulls continuously fed the 7% fNDF diet, with an increase of approximately 26%, whereas the remaining treatments showed intermediate values (*P* = 0.01). However, by d 92, this transition group (12→7% fNDF) displayed the lowest chewing and rumination times among all treatments, representing reductions of approximately 42% and 51%, respectively, compared with the constant 12% fNDF treatment (*P* < 0.01). Regarding idle time, bulls fed the 12% fNDF diet spent approximately 11% and 7% less time idle than bulls fed the 7% fNDF diet during the first period (*P* < 0.01). In the second period, the transition to a reduced-fiber diet (12→7% fNDF) increased idle time by approximately 23% compared with the constant 12% fNDF treatment at d 92, exceeding the other experimental groups (*P* < 0.01).

**Table 8:** Ingestive behavior, intake time, total chewing time, rumination time, and idleness of Angus** × **Nellore bulls fed different fNDF strategies in the feedlot.

Item	Treatment					
	12% fNDF^a^^,^^b^	7% fNDF^b^^,^^c^	12→7% fNDF^b^^,^^d^	7→12% fNDF^b^^,^^e^	SEM^f^	*P-*value
**Intake min/d**						
**21 d**	148.0	132.3	–	–	5.0	0.84
**42 d**	150.0	123.0	–	–	4.7	0.33
**71 d**	127.3	117.6	151.8	126.2	5.1	0.12
**92 d**	132.0	108.4	110.0	121.4	4.5	0.20
**Chewing min/d**						
**21 d**	526.1a	436.5b	–	–	13.3	0.01
**42 d**	465.0a	386.5b	–	–	11.5	0.01
**71 d**	452.0ab	372.3a	468.1b	402.3ab	12.0	0.01
**92 d**	470.0a	350.7b	273.6c	421.5ab	14.1	0.01
**Rumination min/d**						
**21 d**	377.4a	283.6b	–	–	11.9	<0.01
**42 d**	332.6a	263.4b	–	–	10.3	<0.02
**71 d**	324.6	254.6	316.3	276.1	10.7	0.05
**92 d**	338a	242.3b	163.6c	299.2ab	12.7	<0.01
**Idleness min/d**						
**21 d**	884.6b	979.2a	–	–	14.2	<0.01
**42 d**	952.6b	1015.3a	–	–	11.4	<0.01
**71 d**	967.3	1027.6	948.1	1010.7	12.2	0.08
**92 d**	941.4c	1070b	1154.5a	1004bc	14.6	<0.01

a 12% fNDF, constant 12% fNDF throughout the 100 d.

b fNDF, Neutral Detergent Fiber from Forage.

c 7% fNDF, constant 7% fNDF throughout the 100 d.

d 12→7% fNDF, step-down fNDF, from 12% in period 1% to 7% in period 2.

e 7→12% fNDF, step-up fNDF, from 7% in period 1% to 12% in period 2.

f SEM, Standard error of the mean.

### Blood metabolites and inflammatory markers

**Figure 2:**
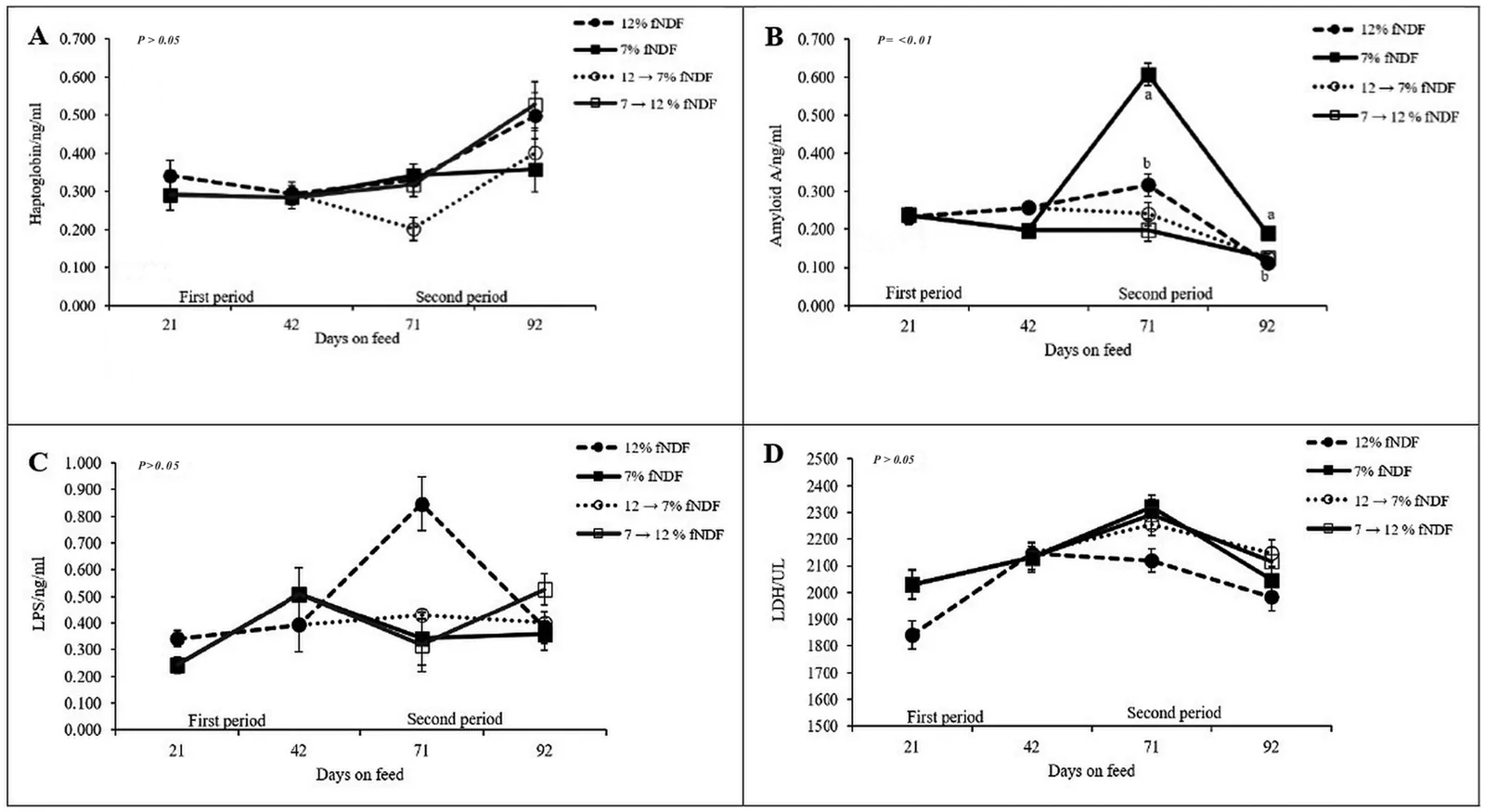
Blood inflammatory markers, (A) haptoglobin, (B) amyloid A, (C) lipopolysaccharide (LPS), (D) lactate dehydrogenase (LDH) of Angus × Nellore bulls fed different fNDF strategies in the feedlot. Means followed by different letters within the same evaluation day differ significantly by Tukey’s test (*P* < 0.05).

Plasma concentrations of LDH, LPS, haptoglobin, and serum amyloid A are presented in Fig. 2. Lactate dehydrogenase, LPS, and haptoglobin concentrations were not affected by dietary treatments (*P* > 0.05). However, during the second period (d 71), bulls continuously fed the 7% fNDF diet exhibited greater serum amyloid A concentrations than the remaining treatments, (*P* < 0.01). By d 92, serum amyloid A concentrations declined across treatments, although bulls continuously fed the 7% fNDF diet remained greater than bulls continuously fed the 12% fNDF diet (*P* < 0.01).

## Discussion

The present study demonstrates that both the level and timing of fNDF supply influence growth performance, starch utilization, and ingestive behavior in Angus × Nellore bulls, particularly during the final 50 days of the feedlot, when bulls are more susceptible to ruminal disturbances (Castillo-Lopez et al. 2014). In contrast to previous studies with purebred Nellore bulls, in which greater forage inclusion frequently increased DMI and ADG (Leme et al. 2003; Caetano et al. 2015; Souza et al. 2026), no performance differences were observed between 7% and 12% fNDF in the present study. These findings support earlier evidence that taurine × zebu crossbred cattle differ from pure *Bos indicus* cattle in ruminal retention time, passage rate, and fermentative dynamics (Phillips et al. 1960; Oliveira et al. 1991; Hegarty 2004). Collectively, these physiological differences reinforce the need for genotype-specific nutritional recommendations.

Souza et al. (2026) reported that Nellore bulls subjected to similar dietary treatments exhibited greater ADG during the first half of the feedlot period when fed 12% fNDF, with no differences over the entire feeding period. In contrast, treatment effects on growth performance in the present study were observed primarily during the final 50 days. Although differences in breed composition may have contributed to these contrasting responses, comparisons between independent studies should be interpreted with caution because the experiments were conducted separately. A direct evaluation of Nellore and crossbred bulls under identical experimental conditions would be required to determine whether breed influences the response to dietary fNDF concentration.

Although the 7% fNDF diet improved ADG during the last 50 d of the feedlot period, it was also associated with greater fecal starch concentration and lower estimated starch digestibility. This response is more likely related to the greater dietary starch concentration of the 7% fNDF diet than to differences in passage rate. As dietary starch supply increases, a greater absolute amount of starch may escape digestion, resulting in increased fecal starch excretion and lower apparent starch digestibility, despite greater dietary energy intake (Krizsan et al. 2010). Therefore, the improved ADG observed with the 7% fNDF diet likely reflects the greater energy density of the diet, although accompanied by less efficient starch utilization. These findings highlight a biological trade-off between maximizing growth performance and optimizing nutrient utilization. Moreover, prolonged feeding of highly fermentable, low-fiber diets may increase the risk of subclinical ruminal dysfunction and greater variation in intake patterns, emphasizing the importance of maintaining adequate physically effective fiber in finishing diets (Allen 1997). From a practical standpoint, nutritionists should consider balancing dietary energy density with adequate fiber supply to maximize animal performance while minimizing losses in starch utilization and maintaining ruminal health during the finishing period.

The temporal modulation of fNDF further clarified this relationship. Increasing dietary fiber from 7% to 12% during the second half of the trial reduced fecal starch concentration and improved estimated starch digestibility without compromising growth performance. This response is consistent with greater chewing activity, enhanced saliva buffering, and a more favorable ruminal environment for microbial starch digestion (Allen 1997). Under the conditions of the present study, increasing fNDF from 7% to 12% during the second half of the feedlot period improved digestive efficiency while maintaining productive performance. These findings should be interpreted within the range of dietary fNDF concentrations evaluated, as responses to greater forage inclusion may differ. Conversely, reducing fiber from 12% to 7% resulted in intermediate digestibility responses, indicating that prior exposure to a was lower-fiber diet may have partially preserved ruminal adaptation after the transition to a more concentrate-rich diet. Similar carryover effects have been reported in cattle exposed to sequential finishing diets with changing roughage concentrations (Pierce et al. 2022).

Crossbred bulls in the present study also appeared to utilize starch more efficiently than purebred Nellore bulls previously evaluated under comparable conditions (Souza et al. 2026). Mean fecal starch values and digestibility coefficients observed here suggest superior starch utilization by the Angus × Nellore genotype. Similar findings were described by Goulart et al. (2020b), who reported lower fecal starch concentrations in crossbred bulls than in purebred Nellore bulls. This may be related to differences in intake behavior, digestive kinetics, pancreatic secretion, or hindgut starch fermentation capacity (Phillips et al. 1960; Oliveira et al. 1991).

Behavioral responses were consistent with the dietary treatments. As expected, diets containing 12% fNDF increased chewing and rumination time during the first period, reflecting the greater structural fiber supply of those diets (Van Soest 1994; Allen 1997; Beauchemin 2018). In contrast, reducing dietary fiber, particularly after the 12→7% transition, decreased rumination and increased idle time, indicating lower masticatory demand and reduced stimulation of rumen motility. These findings reinforce the importance of providing adequate dietary fiber to support normal ingestive behavior and rumen function during the finishing period. Behavioral monitoring, particularly rumination activity, may therefore serve as a practical indicator of dietary adequacy and early ruminal imbalance in feedlot systems (Paudyal 2021).

Despite clear effects on starch utilization and ingestive behavior, reductions in fNDF did not induce consistent systemic inflammatory responses. Plasma LDH, haptoglobin, and LPS concentrations were not altered by treatment, indicating that the lower-fiber diets did not generate measurable systemic inflammation under the conditions of this study. The increase in serum amyloid A observed in bulls continuously fed 7% fNDF during the second period suggests a transient activation of the acute-phase response, despite the absence of changes in plasma LPS, haptoglobin, or LDH. Serum amyloid A is among the earliest and most sensitive acute-phase proteins in cattle and may respond to subtle inflammatory or metabolic challenges before other systemic biomarkers become altered. Therefore, the isolated increase in serum amyloid A may indicate a mild physiological response to prolonged exposure to a highly fermentable, low-fiber diet rather than overt systemic inflammation. This interpretation is consistent with reports showing that highly fermentable diets can induce localized ruminal disturbances and modest acute-phase responses without producing measurable increases in circulating endotoxin or other inflammatory markers (Gozho et al. 2005; Zebeli et al. 2012).

Overall, the present results indicate that this crossbred genotype tolerates reduced fNDF concentrations during finishing and can achieve superior late-feedlot performance under energy-dense diets. However, strategic modulation of fiber supply across the feeding period may represent a more balanced nutritional approach by combining productive efficiency with improved starch utilization and greater ruminal stability.

## Conclusion

Feeding a diet containing 7% fNDF during the second half of the feedlot period improved ADG and feed efficiency but reduced starch utilization. In contrast, increasing dietary fNDF from 7% to 12% during the final 50 d improved starch digestibility without compromising growth performance, suggesting that strategic modulation of dietary fiber may optimize the balance between productive performance and digestive efficiency in feedlot Angus × Nellore bulls. These findings also indicate that fiber recommendations for Angus × Nellore crossbred bulls may differ from those established for purebred Nellore cattle.

## List of abbreviations

ADF: Acid detergent fiber
ADG: Average daily gain
BW: Body weight
DM: Dry matter
DMI: Dry matter intake
DMIg: Dry matter intake for gain
DMIm: Dry matter intake for maintenance
ELISA: Enzyme-linked immunosorbent assay
fNDF: Forage neutral detergent fiber
LDH: Lactate dehydrogenase
LPS: Lipopolysaccharide
NDF: Neutral detergent fiber
(Eg), Mcal/d: Energy required for gain
(Em), Mcal/d: Energy required for maintenance
NEm (Mcal/kg DM): Performance-predicted dietary estimated
NEg (Mcal/kg DM): Performance-predicted dietary estimated
peNDF: Physically effective neutral detergent fiber
SAA: Serum amyloid A
SEM: Standard error of the mean
